# Extracellular Vesicles From Perinatal Cells for Anti-inflammatory Therapy

**DOI:** 10.3389/fbioe.2021.637737

**Published:** 2021-02-05

**Authors:** Anna Cargnoni, Andrea Papait, Alice Masserdotti, Anna Pasotti, Francesca Romana Stefani, Antonietta Rosa Silini, Ornella Parolini

**Affiliations:** ^1^Centro di Ricerca E. Menni, Fondazione Poliambulanza Istituto Ospedaliero, Brescia, Italy; ^2^Department of Life Science and Public Health, Università Cattolica del Sacro Cuore, Rome, Italy; ^3^Fondazione Policlinico Universitario “Agostino Gemelli” IRCCS, Rome, Italy

**Keywords:** perinatal derivatives, secretome, extracellular vesicles, immunomodulation, tissue regeneration

## Abstract

Perinatal cells, including cells from placenta, fetal annexes (amniotic and chorionic membranes), umbilical cord, and amniotic fluid display intrinsic immunological properties which very likely contribute to the development and growth of a semiallogeneic fetus during pregnancy. Many studies have shown that perinatal cells can inhibit the activation and modulate the functions of various inflammatory cells of the innate and adaptive immune systems, including macrophages, neutrophils, natural killer cells, dendritic cells, and T and B lymphocytes. These immunological properties, along with their easy availability and lack of ethical concerns, make perinatal cells very useful/promising in regenerative medicine. In recent years, extracellular vesicles (EVs) have gained great interest as a new therapeutic tool in regenerative medicine being a cell-free product potentially capable, thanks to the growth factors, miRNA and other bioactive molecules they convey, of modulating the inflammatory microenvironment thus favoring tissue regeneration. The immunomodulatory actions of perinatal cells have been suggested to be mediated by still not fully identified factors (secretoma) secreted either as soluble proteins/cytokines or entrapped in EVs. In this review, we will discuss how perinatal derived EVs may contribute toward the modulation of the immune response in various inflammatory pathologies (acute and chronic) by directly targeting different elements of the inflammatory microenvironment, ultimately leading to the repair and regeneration of damaged tissues.

## Extracellular Vesicles

**FIGURE 1:**
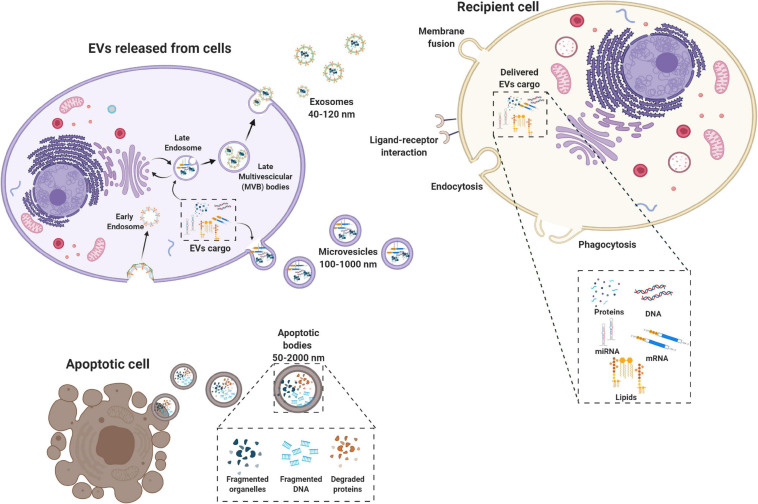
Biogenesis of extracellular vesicles (EVs). EVs are a heterogeneous family of membrane-bound vesicles produced by a donor cell that target a recipient cell. EVs are distinguished into three main subtypes according to their size: microvesicles (100–1,000 nm), exosomes (40–120 nm), and apoptotic bodies (50–2,000 nm). Exosome biogenesis starts from the endosomal system, with the early endosome that transforms into a late endosome, deriving from the inward budding of intraluminal vesicles. EVs can carry many biologically active molecules such as proteins and genetic material (mRNA, miRNA, and DNA), with important immunomodulatory functions. The exosomes produced then can be released by the exocytotic pathway with the fusion between the multivescicular late endosome and the plasma membrane. Microvesicles present a less defined mechanism of biogenesis and release compared to exosomes with different mechanisms that have been identified for the shedding of the MVs depending also on the biological content. Apoptotic bodies are released by dying cells following the blebbing process. EVs are taken up by the recipient cell via different mechanisms including membrane fusion endocytosis, phagocytosis or ligand–receptor interactions. Created with Biorender.com.

Extracellular vesicles (EVs) are membrane-bound vesicles secreted into the extracellular environment by healthy (Raposo and Stoorvogel, 2013) and apoptotic cells (Hristov et al., 2004). Exosomes (exo), microvesicles (MVs) and apoptotic bodies are the three main subtypes of EVs which are distinguished based upon their biogenesis, release pathways, size, content, and function (Zaborowski et al., 2015) (Figure 1). Among the subtypes, the most numerous are exosomes (Rani et al., 2015), whose diameters ranges from 40 to 120 nm. Exosomes form by fusion between multivesicular endosomes and plasma membrane. MVs are between 100 and 1,000 nm in size and bud directly from the plasma membrane. Apoptotic blebs’ size ranges from 50 to 2,000 nm and the bodies are released by dying cells (Zaborowski et al., 2015).

Originally, EVs were thought to serve as a disposal mechanism by which cells eliminate unwanted proteins and other molecules. However, today EVs represent a well-known mechanism of cell-to-cell communication that goes beyond the classical signaling through cell-cell contact and secreted bioactive factors (*i.e.*, cytokines, inflammatory mediators, metabolites, and hormones) (Pitt et al., 2016). Therefore, EVs exert their biological effect through activation of cell signaling by physical ligand/receptor interactions or by fusing with their recipient cells, transferring their contents (miRNAs, mRNAs, proteins, phospholipids, or generally, a morphogen) into the cytosol, and modifying the physiological state of the recipient cells (Figure 1). EVs may also be endocytosed by the target cells or release their bioactive molecules, receptors and genetic information into the extracellular space (Figure 1) (Turturici et al., 2014).

Extracellular vesicles content and mechanism of release differs according to cell origin and it changes in response to fluctuations of physiological states or pathological conditions. During physiological processes EVs can regulate angiogenesis, immune responses, apoptosis, coagulation, cellular homeostasis, and intercellular signaling (Gurunathan et al., 2019). Furthermore, they play a role in the development and progression of diseases (*e.g.*, cancer, neurodegeneration, infections, and cardiovascular disease) (Zaborowski et al., 2015; Kao and Papoutsakis, 2019). EVs can be isolated from a variety of body fluids (*e.g.*, blood, semen, saliva, plasma, urine, cerebrospinal fluid, synovial fluid, malignant and pleural effusions of ascites, bronchoalveolar lavage (BAL) fluid, breast milk, and amniotic fluid) (Rani et al., 2015), and from solid tissues like lung tumors, brain (Hurwitz et al., 2019), and perinatal derivatives, such as placenta (Fitzgerald et al., 2018).

Extracellular vesicles are gaining increasing interest for a broad range of applications, such as potential tools for cancer diagnosis (Rahbarghazi et al., 2019) and therapeutic approaches in regenerative medicine. More specifically, given their role in modulating immune responses, EV-based therapeutics are being developed for the treatment of inflammatory diseases, autoimmune disorders and cancer (Robbins and Morelli, 2014). In order to pursue such applications involving EVs, a better understanding of the immunomodulatory mechanisms through which EVs act, such as deciphering their bioactive cargo and target cells, is needed. The aim of this review is to discuss the current status and advances of EVs from perinatal, or birth-associated, cells isolated from placenta, umbilical cord, and fetal membranes. Perinatal cells and their secretome have demonstrated robust immunomodulatory properties (Mattar and Bieback, 2015; Magatti et al., 2016, 2018, 2019; Abumaree et al., 2017; Lim, 2017; Silini et al., 2017), that have been correlated to their ability to contribute to tissue regeneration (Silini et al., 2017). Hence herein we will discuss the contribution of perinatal-derived EVs to the immune response, with a focus on their ability to promote reparation and regeneration of damaged tissues in the context of acute and chronic inflammation.

## Perinatal Tissues as Sources of Extracellular Vesicles

As mentioned previously, perinatal or birth-associated tissues, refer to tissues that are obtained from term placentas and fetal annexes and more specifically refers to the amniotic membrane, chorionic membrane, chorionic villi, umbilical cord (including Wharton’s jelly), the placental basal plate (including maternal and fetal cells), and the amniotic fluid (Silini et al., 2020).

The fetal membranes enclose the fetus and its surrounding amniotic fluid, forming a highly specialized interface between the mother and the fetus that performs vital functions. The fetal membranes consist of two components: the amnion and the chorion. The amnion, the inner of the two fetal membranes, is a thin, avascular membrane, composed of an epithelial and a mesenchymal layer. The amniotic epithelium is in direct contact with the amniotic fluid and is composed of columnar and cuboidal epithelial cells; the amniotic mesoderm is composed of fibronectin and collagen (type I and III) and hosts rare macrophages and dispersed mesenchymal stromal cells (Silini et al., 2020). Weakly linked but not fused to the amnion, the chorionic membrane consists of a mesodermal region, containing chorionic mesenchymal stromal cells, and a trophoblastic area, rich in proliferating trophoblasts and fibrinoid deposits. In order to maximize exchange surface between mother and fetus tissues, finger-like structures, known as chorionic villi, develop from the outer region of the chorion. The chorionic villi anchor the placenta to maternal endometrium and are involved in fetal-maternal exchange (Silini et al., 2020).

The umbilical cord is an extraembryonic tissue connecting the placenta to the fetus, externally covered by a single epithelial layer of cells, supposed to derive from amniotic epithelium. The structure of umbilical cord is made up of a mucous connective tissue called Wharton’s jelly, enriched in fibroblast-like cells. Within this glycosaminoglycan and collagen-rich matrix are immersed a vein and two umbilical arteries, essential for nutrient, metabolic and gas exchange (Anzalone et al., 2010).

The decidua is the maternal component of placenta and its formation is due to the growth and the proliferation of the functional layer cells of endometrium after implantation. The part of decidua that lies at the site of implantation and interacts with the trophoblasts is defined as decidua basalis, while decidua capsularis refers to the portion of decidua covering the conceptus on the luminal site. The remaining section of endometrium, blending by the fourth month of gestation with the decidua capsularis, is the decidua parietalis that lines the rest of uterus cavity (Abumaree et al., 2016; Turco and Moffett, 2019).

Several cell types can be obtained and expanded from the different regions of the human placenta and the fetal annexes, the most prominent being epithelial cells, mesenchymal stromal cells (MSC), endothelial, and hematopoietic cells, and all of these produce and release EVs.

## Immunomodulatory Properties of Perinatal Cells and Their EVs

The essential role of the placenta in maintaining a state of fetal-maternal tolerance during pregnancy initially suggested that cells derived from gestational tissues may possess intriguing immunomodulatory properties, exploitable for several regenerative medicine applications. Nowadays, the immunomodulatory properties of perinatal cells, especially MSC, have widely been demonstrated (Magatti et al., 2016). Indeed, different *in vitro* studies have demonstrated that perinatal cells target components of the innate and adaptive immune systems, including T and B lymphocytes, macrophages, dendritic cells, neutrophils and natural killer cells.

Specifically, they can suppress the proliferation of T lymphocytes (Magatti et al., 2008; Kronsteiner et al., 2011), and can inhibit the differentiation into Th1 and Th17, causing concurrently the formation of Th2 cells, with an immune regulatory cytokine profile, and the enhancement of regulatory T cells (Pianta et al., 2016; Khoury et al., 2020). In addition, perinatal cells directly interact with B cells, reducing proliferation and plasma cells formation as well as promoting regulatory B cells induction (Che et al., 2012; Magatti et al., 2020). Perinatal cells can also inhibit the migration and maturation of dendritic cells and promote the polarization of monocytes/macrophages toward an anti-inflammatory phenotype (Magatti et al., 2009, 2015; Banas et al., 2013; Croxatto et al., 2014; Abomaray et al., 2015; Abumaree et al., 2019).

In line with this, preclinical studies have shown that administration of perinatal cells or their secretome induces therapeutic effects in many models of inflammatory diseases such as liver (Lee et al., 2010; Manuelpillai et al., 2010, 2012; Jung et al., 2013; Cargnoni et al., 2018), and lung fibrosis (Cargnoni et al., 2009, 2020; Vosdoganes et al., 2011; Murphy et al., 2012; Moodley et al., 2013; Tan et al., 2014, 2017), collagen-induced arthritis (Parolini et al., 2014), experimental autoimmune encephalomyelitis (Parolini et al., 2014; Donders et al., 2015), cerebral ischemia (Lin et al., 2011), and diabetes (Wang et al., 2014; Tsai et al., 2015).

A large body of evidence has demonstrated that these effects are mediated by active molecules secreted by perinatal cells able to affect cell survival, function and repair in host damaged tissues (Gunawardena et al., 2019; Silini et al., 2019). As a matter of the fact, the delivery of conditioned medium (CM), generated from *in vitro* culture of perinatal cells, representing perinatal cell secretome, produced benefits similar to that obtained with parental cells (Cargnoni et al., 2012, 2014; Danieli et al., 2015; Pischiutta et al., 2016; Giampa et al., 2019).

In the last decade, several studies have reported that EVs from perinatal tissues are comparable to the parental cells when transplanted in several preclinical models of inflammatory mediated diseases such as wound healing (Li et al., 2016; Zhao et al., 2017), pulmonary fibrosis (Tan et al., 2018), hepatic fibrosis (Alhomrani et al., 2017); bronchopulmonary dysplasia (BPD) (Chaubey et al., 2018; Willis et al., 2018), liver failure (Jiang et al., 2019; Yao et al., 2019), vascular repair (Spinosa et al., 2018; Wei et al., 2019), renal injury (Zou et al., 2014, 2016), neurodegenerative diseases (Ding et al., 2018; Ma et al., 2019; Romanelli et al., 2019; Thomi et al., 2019), autoimmune diseases (Bai et al., 2017; Mao et al., 2017), and Duchenne muscular dystrophy (Bier et al., 2018). Furthermore, EVs have the advantage of being a cell-free therapy and therefore with reduced risks associated with the transplantation of live cells.

In relation to the therapeutic utility of perinatal EVs assessed in the above cited preclinical studies, there are five clinical trials applying EVs from perinatal cells reported in the ClinicalTrials.gov database and one reported in Chinese Clinical Trial Registry. They are phase I studies with the primary endpoint to establish the safety of the treatment. One of these (NCT03437759), will apply exosomes from human UC-MSCs to large and refractory macular holes (MHs). Another study (NCT04213248), explores whether the local delivery of exosomes from UC-MSCs is able to reduce dry-eye symptoms in patients with chronic Graft Versus Host Diseases (cGVHD). Exosomes from UC-MSCs will also be used to treat multiple organ dysfunction syndrome after surgical ascending aortic replacement (NCT04356300). Exosomes from another source, amniotic fluid, are under evaluation to treat, in combination with ultrasound therapy, depression, anxiety, and neurodegenerative dementia in patients resistant to any pharmacological treatment (NCT04202770).

Finally, two more trials, one using EVs derived from human amniotic fluid (NCT04384445) and the other using exosomes from umbilical cord MSCs (ChiCTR2000030484 study, from Chinese Clinical Trial Registry), will assess the ability of these treatments to suppress cytokine activation and any incidence of associated adverse events, in subjects suffering from COVID-19 infection with severe acute respiratory syndrome.

### Impact of Perinatal EVs on Cells of the Myeloid Lineage

It is now widely recognized that the therapeutic effect of perinatal cells is largely mediated via secretion of bioactive factors rather than cell–cell interactions (Silini et al., 2019). Many studies have suggested the MSC EVs modulate inflammation and contribute to tissue regeneration (Fatima et al., 2017), however, the exact mechanism by which perinatal derived/secreted factors regulate the immune response is still unknown. Importantly, several recent papers focused their attention on EVs from perinatal MSC, highlighting their potential for a cell-free therapy (Robbins and Morelli, 2014; Phinney and Pittenger, 2017).

**FIGURE 2:**
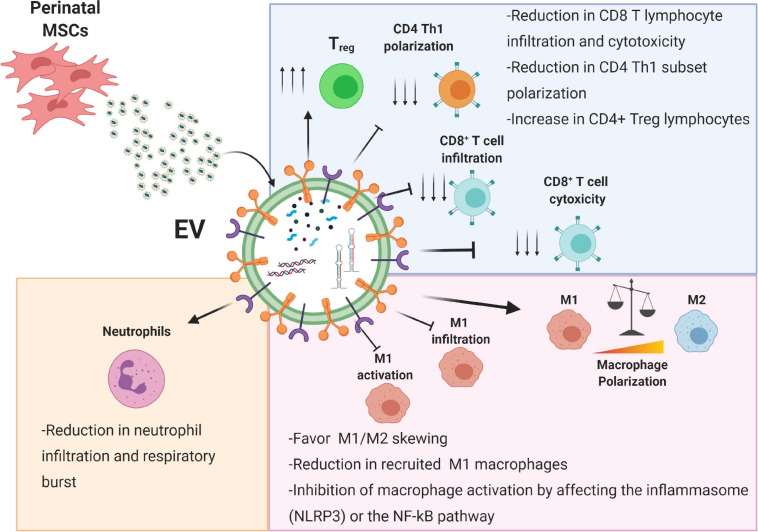
Immunomodulatory effects of perinatal-MSC extracellular vesicles (EVs). The molecular content of perinatal MSC-EVs represented by proteins, lipids, and nucleic acids can strongly affect both the innate (neutrophils and macrophages) and adaptive immune response (T lymphocytes). Treg, T regulatory cell; Th1, T helper 1 cell; M1, macrophage type 1; M2, macrophage type 2; NLRP3, NLR family pyrin domain containing 3; NF-κB, nuclear factor kappa B. Created with Biorender.com.

Innate immune cells, including neutrophils, NK cells, and phagocytic cells such as macrophages and dendritic cells, are the first cells to initiate an immune response against a potential pathogen, clear from residual apoptotic or necrotic cells, and remodel the extracellular matrix to prepare the “scenario” pivotal to the subsequent healing steps (White and Mantovani, 2013; MacLeod and Mansbridge, 2016). Perinatal EVs have been shown to interact with various innate immune cells (Figure 2). In the following sections we describe the interactions between perinatal EVs and immune cells (summarized in Figure 2) and identify several key molecules involved in these interactions (summarized in Table 1).

**TABLE 1:** Summary of perinatal EVs mechanisms of action and biological effects.

**Cell type**	**Model**	**Target cells**	**Key molecules/Molecular mechanism**	**Biological effect**	**References**
hUC-MSC	Rat hepatic ischemia-reperfusion injury	Hepatocytes, Neutrophils	Mitochondrial manganese superoxide dismutase Mn(SOD), anti-oxidant activity	Decreased expression of inflammatory cytokines in hepatic tissue and serum, decreased hepatocyte necrosis	Yao et al. (2019)
hUC-MSC	Mouse model of acute liver failure; RAW 264.7 macrophages *in vitro* activated with LPS	Macrophages	Inhibition of NLRP3 inflammasome complex expression	Reduced caspase 1 and inflammatory cytokines (IL-1β and IL-6)	Jiang et al. (2019)
hUC-MSC	Mouse model of acute burn	Macrophages	miR-181c downregulation of TLR-4 receptor expression with consequent downregulation of NF-κB/p65 pathway	Reduction of M1 macrophage activation	Li et al. (2016)
hUC-MSC	Mouse model of aortic aneurysm	T lymphocytes	miR-147	Decreased expression of inflammatory cytokines, reduction of lymphocytes infiltration, improved aortic diameter, and elasticity	Spinosa et al. (2018)
hUC-MSC (LPS primed)	Wound healing model in diabetic rat	Macrophages	miR-let-7b down regulation of TLR4/NF-κB/STAT3/AKT signaling pathway	Promotion of M2 macrophage activation, enhanced diabetic cutaneous wound healing	Ti et al. (2015)
hUC-MSC (IL-1β primed)	Mouse model of sepsis	Macrophages	miR-146a	Increased polarization of macrophages toward the M2 anti-inflammatory subset	Song et al. (2017)
hUC-MSC	Mouse and rat spinal cord injury model	Macrophages	-	Reduction of inflammatory cytokines and M2 polarization	Romanelli et al. (2019); Sun et al. (2018)
hUC-MSC	Mouse model of inflammatory bowel disease	Macrophages	-	Reduced macrophage recruitment and reduced inflammation	Mao et al. (2017)
hUC-MSC	Mouse model of retinal laser injury	Macrophages	-	Downregulation of MCP1 and macrophage infiltration	Yu et al. (2016)
hUC-MSC	Rat model of experimental autoimmune uveoretinitis	CD4 T lymphocytes, NK cells, neutrophils and macrophages	-	Reduced infiltration of inflammatory immune cells	Bai et al. (2017)
hUC-MSC	Synthetic vascular grafts in rat model of hyperlipidemia	Macrophages	-	Reduced macrophage infiltration and enhanced M2 macrophage polarization, associated with reduced thrombosis and vascular intimal hyperplasia	Wei et al. (2019)
hUC-MSC	Alzheimer mouse model	Microglia	-	Reduced number of M1 microglial cells, promotion of M2 microglial polarization associated with reduced neuroinflammation and B amyloid deposition	Ding et al. (2018)
hUC-MSC	Human *in vitro* purified T lymphocytes	T lymphocytes	Adenosine signaling through CD73 expression	Reduced T lymphocyte activation	Kerkelä et al. (2016)
hUC-MSC	Mouse model of contact hypersensitivity	T lymphocytes	STAT1 signaling pathway inhibition	Reduced cytotoxic and Th1 lymphocyte infiltration and induced Treg with reduced tissue swelling	Guo et al. (2019)
hUC-MSC	Human *in vitro* purified T lymphocytes	T lymphocytes	-	Reduced T lymphocyte proliferation and inflammatory cytokine production	Monguió-Tortajada et al. (2017)
hUC-MSC	GVHD mouse model	T lymphocytes	-	Reduced CD8 cytotoxic T lymphocyte and reduced inflammatory cytokines in serum, alleviated GVHD manifestations and reduced mortality of the recipient mice	Wang et al. (2016)
hUC-MSC (β2 microglobulin deficient)	Rat model of myocardial infarction	CD8 T lymphocytes	miR-24	Reduced CD8 T lymphocyte cytotoxicity with improved preservation of cardiac function after myocardial infarction	Shao et al. (2020)
hUC-MSC (primed with TGFβ and IFNy)	*In vitro* study on human PBMC	Treg	IDO1	Increased Treg polarization	Zhang et al. (2018)
hUC-MSC	*In vitro* study on hUC-MSC and hCB-MSC	miRNA profiling	miR-a25b, miR26a, miR145, miR181c-5p, miR-Let7e, miR-Let7c, miR-Let7f, and miR.106a	miRNAs with immunoregulatory functions	Meng et al. (2017)
hUC-MSC	Rat model of renal ischemic reperfusion injury	NK cells	CXC3L1, TLR-2	Reduced CXC3L1 and TLR-2 expression with consequent reduced NK cell recruitment	Zou et al. (2016)
hWJ-MSC	Mouse model of acute kidney injury	Macrophages	-	Decreased CXCL3 ligand 1, reduced macrophage and T lymphocyte infiltration, and reduced renal injury	Zou et al. (2014)
hWJ-MSC	Perinatal brain injury rat model	Microglia	TLR4/CD14 signaling pathway inhibition	Reduced expression of inflammatory cytokines by microglial cells	Thomi et al. (2019)
hWJ-MSC	Mouse model of hyperoxia-induced bronchopulmonary dysplasia (BPD)	Neutrophils	Tumor necrosis factor alpha-stimulated gene-6 (TSG-6)	Suppression of hyperoxia-induced neutrophil accumulation in bronchoalveolar lavage (BAL) with improvement of BPD pathology	Chaubey et al. (2018)
canine WJ-MSC	*In vitro* study with canine PBMC	CD4 T lymphocytes	TGF-β1/Adenosine signaling	Reduced CD4 T lymphocyte proliferation	Crain et al. (2019)
hWJ-MSC	Hyperoxia-induced bronchopulmonary dysplasia	Macrophages	-	Reduced M1 macrophage lung infiltration, enhanced M2 polarization	Willis et al. (2018)
hAF-MSC (primed with IFNy)	*In vitro* co-culture with PBMC; mouse model of allogeneic skin graft transplantation	Treg	IDO1	Increased number of Treg cells promoting *in vivo* allograft survival	Romani et al. (2015)
hAF-MSC	*In vitro* THP1 macrophage cell line; mouse model of monoiodoacetate induced osteoarthritis	Macrophages	-	Promoted skewing of THP1 committed M1 macrophages toward M2 subset. *In vivo* increased pain tolerance and improved histopathological score	Zavatti et al. (2020)
hAF-SC	*In vitro* and *in vivo* models of skeletal muscle atrophy (HSA-Cre, SmnF7/F7 mice)	B lymphocytes	-	Reduced B lymphocyte maturation and decreased skeletal muscle inflammation associated with enhanced muscle strength and survival	Balbi et al. (2017)
hAEC	Mouse model of CCl4 induced liver fibrosis	Macrophages	-	Reduced liver fibrosis through promotion of M2 macrophage polarization	Alhomrani et al. (2017)
hAEC	Bleomycin-induced pulmonary fibrosis	Macrophages/T lymphocytes	-	Reduced lung fibrosis through promotion of M2 macrophage polarization and reduction of T cell infiltration	Tan et al. (2018)

*hUC-MSC, human umbilical cord mesenchymal stromal cells; hAF-SC, human amniotic fluid stem cells; hAF-MSC, human amniotic fluid mesenchymal stromal cells; hWJ-MSC, human Wharton’s jelly mesenchymal stromal cells; IDO1, indoleamine-2,3-dioxygenase-1; LPS, lipopolysaccharide; TLR-2/4, Toll like receptor 2 or 4; CXC3L1, chemokine (C-X3-C motif) ligand 1; GVHD, acute graft-vs-host disease; NK, natural killer cells; MCP-1, monocyte chemotactic protein-1, PBMC; human peripheral blood mononuclear cells.*

#### MSC-EVs Interfere With Infiltration and Accumulation of Neutrophils

Several studies have shown the ability of perinatal EVs to act on neutrophils. For example, in a rat model of hepatic ischemia-reperfusion injury, IV injection of EVs from human umbilical cord MSC (hUC-MSC) reduced serum biomarkers of liver injury (ALT, AST, and ALP), hepatic necrosis and hepatocyte apoptosis (Yao et al., 2019). These effects were mediated by EVs ability in reducing neutrophil hepatic infiltration, suppressing neutrophil respiratory burst (*in vitro* evidence) and in decreasing the expression and the levels of inflammatory cytokines (IL-1β, IL-6, and TNF-α) in hepatic tissues and serum, respectively. The authors suggested that the hepatic protective effects of hUC-MSC EVs may be mediated through the vesicular secretion of a crucial enzyme with anti-oxidant action (MnSOD) (Yao et al., 2019). However, no mechanism has been explored to explain EVs action on neutrophil recruitment.

Exosomes derived from Wharton’s jelly MSC (hWJ-MSC) of premature neonates, injected intraperitoneally in a mouse model of hyperoxia-induced BPD, decreased lung inflammation, alveolar structural alterations, endothelial cell damage and demyelination in the brains (Chaubey et al., 2018). Interestingly, exosome treatment specifically suppressed the hyperoxia-induced neutrophil accumulation in BAL while it did not affect BAL macrophage levels. In the same study, a group of BPD animals received either the CM (hWJ-MSC-CM) or the exosome-depleted fraction of CM, and while both hWJ-MSC-CM and hWJ-MSC-exosome treatments improved BPD pathology, the exo-free fraction did not, suggesting that the exosome fraction is responsible for the beneficial effects observed. Interestingly, the authors detected the presence of tumor necrosis factor alpha-stimulated gene-6 (TSG-6) in exosomes and in CM and observed that the use of a TSG-6 neutralizing antibody or of exosomes obtained from TSG-6 siRNA knockdown hWJ-MSC abolished the therapeutic effects.

#### MSC-EVs Affect Macrophage Activation and Polarization

Human umbilical cord MSC EVs have been reported to differently affect the activity of inflammatory macrophages, by modulating their activation (Li et al., 2016), favoring a skewing toward anti-inflammatory M2 macrophages, or modulating their recruitment to the injured site of inflammation (Yu et al., 2016; Willis et al., 2018; Wei et al., 2019). Jiang et al. (2019) reported the capacity of UC-MSC-derived exosomes to directly inhibit M1 macrophage activation in a mouse model of acute liver failure by inhibiting the expression of the NLRP3 inflammasome complex and the production of inflammatory cytokines. These findings were also confirmed in a cell line of LPS-stimulated RAW 264.7 macrophages, where the administration of UC-MSC exosomes was able to reduce the expression of NLRP3, caspase 1 as well as of the inflammatory cytokines IL-1β and IL-6 (Jiang et al., 2019). In another mouse model of acute burn, administration of the UC-MSC-derived exosomes strongly reduced the activation of M1 macrophages by impairing the NF-κB/p65 pathway, consequently reducing the expression of TNFα, IL-1β and increasing the amount of IL-10 produced and released (Li et al., 2016). As a matter of fact, *in vitro* studies performed in a cell line of mouse macrophages identified a specific miRNA, miR-181c, as the one responsible for the effect observed. Indeed, this miRNA directly affects the expression of toll-like receptor 4 (TLR4), whose downregulation directly impacts NF-κB/p65 pathway activation (Li et al., 2016).

Importantly, EVs can regulate the phenotype polarization of M1 toward M2 macrophages thus reducing the release of inflammatory cytokines. Using the human monocytic cell line THP1, Ti et al. (2015) reported that exosomes from LPS-primed UC-MSC were able to affect the M1/M2 skewing and enhance the expression of miR-Let7b. The overexpression of this miRNA can directly impact M1 polarization favoring instead a skewing toward the M2 subset by downregulating the TLR4/NF-κB/STAT3/AKT signaling pathway which is essential for the regulation of macrophage plasticity (Ti et al., 2015). Comparable effects were observed when UC-MSC were primed with the inflammatory cytokine IL-1β. Indeed, researchers observed that the priming was able to trigger an increase in the total amount of miRNA-146a molecules contained in the UC-MSC EVs resulting in a strong polarization of bone marrow-derived macrophages toward M2 macrophages both *in vitro* and in an animal model of sepsis (Song et al., 2017). Similarly, IV injection of EVs and exosomes from hUC-MSCs was able to reduce inflammatory cytokines and induce M2 macrophage polarization with a concomitant improvement of motor function in models of spinal cord injury induced in mice (Sun et al., 2018) and in rats (Romanelli et al., 2019), respectively.

Analogous findings were observed by Zavatti et al. (2020) in a murine model of monoiodoacetate-induced osteoarthritis. The authors report the capability of amniotic fluid-derived MSC (hAF-MSC) to directly affect the differentiation of the THP1 cell line toward the M1 subset (Zavatti et al., 2020). Indeed, exposure of THP1 committed M1 macrophages to hAF-MSC EVs, strongly reduced the expression of canonical M1 markers like iNOS, the expression of the co-stimulatory molecules CD86 and IL1R favoring instead the acquisition of feature typical of M2 macrophages like the expression of arginase, CD163 and TGFβ (Zavatti et al., 2020). Moreover, the authors reported the ability of hAF-MSC-EVs to increase pain tolerance and improve histopathological scores (Zavatti et al., 2020).

#### MSC-EVs Prevent Inflammatory Immune Cell Recruitment

Furthermore, UC-MSC EVs can also affect the inflammatory microenvironment by preventing the migration of pro-inflammatory M1 macrophages (Zou et al., 2014; Yu et al., 2016; Bai et al., 2017). Injection of WJ-MSC EVs in an animal model of acute kidney injury was able to reduce the renal cell apoptosis by reducing the infiltration of inflammatory macrophages thus improving the survival and proliferation of the renal cells. This effect was due to the down-modulation of CXCL3 ligand 1, a chemotactic factor essential for monocyte and T lymphocyte recruitment in the injured area (Zou et al., 2014). Moreover, WJ-MSC EVs were able to strongly impact the total number of CD68^+^ immune cells recruited, correlating the potential therapeutic effect of the treatment to the reduced amount of infiltrating inflammatory cells (Zou et al., 2014). hUC-MSC exosomes were also able to reduce acute inflammatory macrophage hepatic infiltration and related liver injury induced by intraperitoneal injection of LPS toxin (Jiang et al., 2019). hUC-MSC exosomes also reduced macrophage infiltration in colon tissues after injection in mice with dextran-induced inflammatory bowel disease (Mao et al., 2017).

In another study, Yu et al. (2016) reported the capacity of UC-MSC EVs to reduce the migration and infiltration of inflammatory cells in a mouse model of retinal laser injury. This effect was due to the reduction of MCP1, TNF-α, and ICAM1. Importantly, these findings were confirmed also in *in vitro* experiments where the treatment with UC-MSC EVs was able to reduce the expression of MCP1 thus positively impacting the amount of heat-induced apoptosis or death of retinal cells (Yu et al., 2016). These findings suggested that UC-MSC EVs exert their protective effect, at least partially, through regulation of MCP-1 and macrophage infiltration. Exosomes from hUC-MSC were also able to inhibit the autoimmune response in a rat model of experimental autoimmune uveoretinitis (EAU) (Bai et al., 2017). hUC-MSC exosomes reduced the amount of CD4^+^ T cells, neutrophils, NK cells and macrophages infiltrating the retina.

Recently, Wei et al. (2019) used EVs from hUC-MSC to functionalize synthetic vascular grafts in order to reduce inflammatory-induced thrombosis and vascular intimal hyperplasia occurring in hyperlipidemic rats. hUC-MSC-derived EVs enhance the patency of vascular grafts by reducing macrophage infiltration and inducing M2 polarization. In another study performed in a mouse model of aortic aneurysm formation, mice treated with UC-MSC EVs displayed decreased expression of inflammatory cytokines and in parallel reduced lymphocyte infiltration thus improving aortic diameter and elasticity through the activity played by miR-147 (Spinosa et al., 2018).

Furthermore, EVs from human amniotic membrane epithelial cells (hAEC) were able to reduce macrophage infiltration in the liver and lung parenchyma in a model of CCl4-induced liver fibrosis (Alhomrani et al., 2017) and in a model of bleomycin-induced pulmonary fibrosis (Tan et al., 2018). hAEC-EVs were also able to promote M2 macrophage polarization (Alhomrani et al., 2017; Tan et al., 2018), while reducing pulmonary T cell infiltration (Tan et al., 2018). Interestingly, exosomes from WJ-MSC were also able to reduce macrophage lung infiltration when IV injected in a model of hyperoxia-induced BPD (Willis et al., 2018).

#### MSC-EVs Modulate the Microglial Inflammatory Response

Tissue resident macrophages of the central nervous system, microglia, play a pivotal role in modulating the inflammatory response (Lenz and Nelson, 2018). Thomi et al. (2019) reported that the administration of WJ-MSC-EVs was able to modulate the microglia response by interfering with the TLR4/CD14 signaling pathway thus dampening the expression of inflammatory cytokines such as TNF-α and IL-6 but without affecting the inflammasome pathway. Furthermore, in a transgenic mouse model of Alzheimer’s disease exosomes derived from hUC-MSC ameliorated spatial learning and memory function and, in parallel, reduced the number of inflammatory M1 microglial cells and increased levels of M2 immunomodulatory microglia (Ding et al., 2018). In addition, reduced levels of inflammatory cytokines (IL-1β and TNF-α) and increased of anti-inflammatory ones (IL-10 and TGF-β) were found in peripheral blood and in brains of mice treated with hUC-MSC exosomes.

### Impact of Perinatal EVs on Cells of the Lymphoid Lineage

#### Perinatal MSC-EVs Influence T Lymphocyte Activation and Polarization

In addition to the effect observed on innate immune cells, several groups have also shown that perinatal EVs modulate the adaptive immune response, as well as the infiltration of T lymphocytes in the inflamed tissues. Crain et al. (2019) reported that WJ-MSC EVs were able to inhibit CD4 T lymphocyte proliferation in a dose-dependent manner. The observed effect was possibly due to the high amount of TGFβ1 delivered by the EVs. Indeed, the administration of TGFβ1 neutralizing antibodies and TGFβ1R antagonist reverted the efficacy of the WJ-MSC EVs. Similar findings were also obtained when adenosine signaling was blocked, thus suggesting that the mechanism of action of WJ-MSC-EVs was based on the activation of these two axes (Crain et al., 2019). As a matter of fact, similar findings were observed also by Kerkelä et al. (2016), who reported the capacity of UC-MSC EVs to affect the extracellular microenvironment through the production of adenosine by the CD73 ectonucleotidase expressed on the surface of the EVs. The authors reported how CD39 expressed by the T lymphocyte synergizes with CD73 expressed on the surface of the exosomes to convert the extracellular ATP in ADP, AMP, and finally in adenosine, thus inhibiting the immune response (Kerkelä et al., 2016).

hUC-MSC EVs have been also used to treat immune-dysregulated diseases such as allergic dermatitis characterized by an excessive antigen-specific T cell reaction (Guo et al., 2019). Application of hUC-MSC EVs in a mouse model of contact hypersensitivity (CHS) reduced ear swelling and ear leukocyte infiltration, reduced the percentages of IFN-γ producing CD8^+^ and CD4^+^ T cells, increased the levels of T regulatory (Treg) cells in the cervical lymph nodes, and finally, decreased serum levels of IFN-γ and TNF-α while increasing those of IL-10 (Guo et al., 2019). The authors suggested that the immunomodulatory action of hUC-MSC EVs may be mediated by their ability to suppress STAT1 activation (Guo et al., 2019).

The ability of UC-MSC EVs to affect T lymphocyte activation and proliferation was also observed in a comparison article aimed to determine whether the different fractions of the secretome display distinct immunomodulatory properties. Indeed, one study that compared the results obtained from the complete CM, the ultracentrifuged pellet, the non-EV fraction, and the EV fraction isolated by size-exclusion chromatography (SEC) (Monguió-Tortajada et al., 2017). Importantly, the authors reported that EVs do not induce monocyte polarization or cytokine secretion, but the non-EV fraction is able to trigger the expression of two M2 markers namely CD163 and CD206, while at the same time enhance the production of the inflammatory cytokine TNF-α (Monguió-Tortajada et al., 2017). Moreover, the SEC-purified EV fraction was the only fraction able to inhibit T lymphocyte proliferation and inflammatory cytokine production *in vitro*, resembling the effect of parental UC-MSC. Furthermore, the other fractions triggered the polarization of polyclonally stimulated T cells toward the inflammatory Th17 subset (Monguió-Tortajada et al., 2017). Additionally, the ability of UC-MSC EVs to affect Th subset polarization was also reported by Wang et al. (2016). Indeed, the authors highlighted how UC-MSC-EVs can trigger the conversion of inflammatory Th1 subset toward the Th2 subset by the downregulation of the pro-inflammatory cytokines TNFα and IFNγ, and instead trigger the expression of IL-10 and IL-4. Furthermore, when inoculated in a mouse model of graft versus host disease, UC-MSC EVs were able to reduce the serum level of inflammatory cytokines IL-2, TNFα, and IFNγ, increase the amount of IL-10, and reduce the absolute number of cytotoxic CD8 T lymphocytes (Wang et al., 2016).

In line with this, another study reported that EVs isolated from the B2 microglobulin negative fraction of UC-MSC lack of the capacity to trigger the activation of cytotoxic CD8 T lymphocytes. Moreover, authors observed an enrichment of miR-24 promoting survival of cardiomyocytes by targeting Bim. As a matter of fact, the authors highlighted the capacity of UC-MSC EVs to counteract fibrosis induced by the TGFβ pathway in an *in vivo* model of myocardial infarction in rats (Shao et al., 2020). These findings were also confirmed by Guo et al. (2019), who reported that, both *in vitro* and in an *in vivo* model of allergic contact dermatitis (ACD), UC-MSC-EVs were able to reduce the total amount of cytotoxic CD8 IFNγ + T lymphocytes, and inhibit the polarization of the inflammatory CD4 Th1 subset and instead foster the Treg polarization. In a different study, internalization of the EVs was able to reduce the STAT1 protein level thus affecting the transcriptional polarization toward the inflammatory and cytotoxic T lymphocyte subsets (Guo et al., 2019).

#### Perinatal EVs Impact Treg Polarization and T Lymphocyte Recruitment

Perinatal EVs can also affect Treg polarization. In this context, Zhang et al. (2018) reported how UC-MSC primed with TGFβ and IFNγ for 72 h were able to enhance Treg polarization, putatively through upregulation of IDO molecules.

Similarly, EVs isolated from primed amniotic fluid MSC were able to induce the polarization and expansion of CD4 Treg cells. Romani et al. (2015) reported that pre-treatment with IFNγ was able to strongly increase the amount of IDO1 conveyed by the EVs. These *in vitro* findings were confirmed in a mouse model of allogeneic skin graft transplantation, where the treatment with hAF-MSC EVs induced an increase in the number of Treg cells in the draining lymph nodes of recipient mice (Romani et al., 2015). Converserly, Balbi et al. (2017) highlighted how EVs isolated from hAF-SC were not able to affect the proliferation of human PBMC activated with different stimuli. Furthermore, they did not observe any significant effect in the polarization of CD4 Treg cells, while a significant reduction on B cell maturation was detected (Balbi et al., 2017). Finally, several studies also reported the ability of EVs isolated from UC-MSC and hAEC to inhibit the recruitment of inflamed T lymphocytes. Indeed, in a mouse model of uveoretinitis Bai et al. (2017) reported a reduction of IFN-γ- and IL-17-producing CD4^+^ T cells in the damaged area. However, these findings were associated neither with a diminished T cell proliferation nor with increased cell apoptosis, but rather with the ability of hUC-MSC exosomes to inhibit T cell migration (Bai et al., 2017). Similar results were reported also by Tan et al. (2018), highlighting the potential immunomodulatory effect of hAEC EVs, able not only to induce polarization of monocytes toward anti-inflammatory M2 macrophages, but also to reduce the infiltration of T lymphocytes in a mouse model of bleomycin induced pulmonary fibrosis. Besides T-cells, *in vitro* and *in vivo* studies have reported the capacity of the exosomes isolated from UC-MSC to affect other lymphoid cells, such as the migration of NK cells by down modulating the expression of the C-X3-C motif chemokine ligand-1 (CX3CL1) and toll-like receptor-2 (TLR-2), thus affecting NK cell recruitment (Zou et al., 2016).

## miRNAs Regulate the Immune Modulatory Properties of Perinatal EVs

Many of the observed effects can be in part attributable to the presence of bioactive molecules like the miRNAs. As a matter of fact, among the bioactive factors contained in EVs, miRNAs have emerged as one of the main effectors in regulating several biochemical and transcriptional pathways such as proliferation, differentiation, inflammation, metabolism, and apoptosis (He and Hannon, 2004). Indeed, several miRNAs have been associated with the modulation of T lymphocytes being able to boost or dampen their activation and polarization in order to maintain homeostasis (Rodríguez-Galán et al., 2018). At present, only a few studies have attempted to characterize the miRNA profile of the EVs isolated from perinatal MSC (Fang et al., 2016; Balbi et al., 2017; Meng et al., 2017; Zou et al., 2018). Importantly, these studies reported the presence of a few specific and highly expressed miRNAs, previously described for their immunomodulatory potential, such as miR-16, miR-Let7-c, miR-181a, miR-125b, miR-26a, miR-145, miR-181c-5p, miR-Let-7e, miR-Let7-c, miR-Let-7f, and miR-106a (Meng et al., 2017). Moreover, UC-MSC EVs express high levels of miR16 and miR-Let-c (Zou et al., 2018). These two miRNA have been reported as directly targeting the 3′UTR of mTOR and RICTOR consequently reducing mTOR signaling and triggering Treg cell induction (Marcais et al., 2014). Since all these miRNAs impact the function of T lymphocytes, they represent possible mechanisms of action for the effects of EVs described so far.

The effects of perinatal EVs/exosomes may be mediated by a variety of miRNA that alter the activity of macrophages. For example, Ti et al. (2015) suggested that exosomes from LPS-primed hUC-MSC can affect macrophage plasticity through TLR4/NF-κB/STAT3/AKT regulatory signaling pathway via let-7b miRNA (Ding et al., 2018). The authors suggested that let-7b may activate AKT pathway which, in turn, suppresses macrophage TLR4/NF-κB activation and the resulting inflammatory response. TLR4/CD14 signaling pathway was affected also through exosomal delivery of miR-181c (Yao et al., 2019) and by the treatment with hWJ-MSC-exosomes which possibly act via the exosomal shuttle of miR-146a/b (Ma et al., 2019).

## Conclusion, Challenges and Future Directions

The studies performed until now have on one hand demonstrated the attractive therapeutic potential of EVs derived from perinatal cells, but, on the other hand, they uncover the limitations of our knowledge and the need to solve many critical aspects before the translation of these products as therapeutic tools in clinic.

A common observation that should be outlined is the great heterogeneity in methods/techniques applied to obtain EVs/exosomes. Lack of standardized procedures does not assure the reproducibility, purity and maintenance of EV functional properties (Théry et al., 2018). The most applied EV-isolation methods in these papers are ultracentrifugation and precipitation kits each of which differ for recovery and specificity (Théry et al., 2018). Different methods of EV quantification have been applied, the most used is the total protein amount, some report the particle numbers (Alhomrani et al., 2017; Bier et al., 2018; Li et al., 2020), one the RNA concentration (Li et al., 2016) and others the injection volume (Alhomrani et al., 2017; Willis et al., 2018).

Another parameter that makes comparison difficult is referred to the conditioned media from which EVs/exosomes are isolated. The detailed preparation protocol for CM is often omitted in the publications, even if it is well known that culture conditions (cell passage, cell density, culture volume, culture medium, culture duration, etc.) affect cell functions as well as cell EV/exosome production. It is important to consider that supplements, such as serum, used in cell culture media, may contain EVs therefore ultimately affecting readouts. Although the use of serum-free medium or EV-depleted medium is strongly advisable, some studies use medium with serum (Li et al., 2016). Moreover, in order to exclude any possible contribution of the medium itself to EV composition, negative controls are needed. In this case, negative control means the “material” obtained from culture medium not conditioned by cells but processed in the same way as CM (Théry et al., 2018). Very few studies include this negative control in the planned treatment groups (Ding et al., 2018), while often this control is incorrectly replaced with a group treated with PBS, representing the vehicle in which EVs/exosomes are usually dissolved.

An important aim of some of the above reported studies is to compare the therapeutic efficacy of EVs/exosomes with respect to that of parental cells (Bier et al., 2018; Romanelli et al., 2019; Yao et al., 2019; Zavatti et al., 2020). However, this outcome is compromised by the lack of equivalent doses (Bier et al., 2018; Yao et al., 2019), differences in frequency and timing of delivery (Zavatti et al., 2020), and very few studies testing more than one dose (Sun et al., 2018).

Another important point, but that only a few studies have addressed (Alhomrani et al., 2017; Chaubey et al., 2018), is to establish whether treatment with EVs/exosomes is advantageous (for example, in terms of efficacy, effect duration, and tissue distribution) with respect to the treatment with CM in toto or with EV-depleted CM, to rationalize the time and money consuming procedure to isolate EVs from CM.

Some studies tried to approach another important point: the possible functional differences among EVs/exosomes derived from MSC from different sources and contrasting results have been reported. Willis et al. (2018) observed that, when IV injected in a murine model of hyperoxia-induced BPD, exosomes from bone marrow-derived MSC (BM-MSC) exerted therapeutic effects comparable to exosomes from WJ-MSC, while no benefits were observed after treatment with exosomes from human dermal fibroblasts. Instead, other authors found that in a model of bleomycin-induced pulmonary fibrosis exosomes from human lung fibroblasts show some of the anti-inflammatory effects observed when hAEC exosomes are used (Tan et al., 2018). In contrast with Willis et al. (2018); Bier et al. (2018) found that, in a model of Duchenne muscular dystrophy, BM-MSC-derived exosomes, unlike placenta-derived, did not ameliorate the pathology.

Another major point scarcely studied is the ability of EVs/exosomes to home to injury sites. Ma et al. (2019) injected fluorescent-labeled hUC-MSC EVs in rat tail vein and 24 h after injection EVs were found in the muscle lesioned by sciatic nerve transection, suggesting that EVs may maintain the tropism of parental cells.

Currently, there are a few ongoing clinical trials using MSC-derived EVs/exosomes. An updated search (December 2020) in the database of NIH https://clinicaltrials.gov/using the key words “MSC exosomes OR MSC extracellular vesicles” resulted in 13 clinical trials, most of these using exosomes derived from BM-MSC or from adipose MSC, and only 6, as mentioned previously, regarded the application of perinatal EVs.

Even considering the ongoing clinical trials, a concerted effort is still required to standardize perinatal EVs. Consortiums such as the COST SPRINT Action (CA17116), which aims to approach consensus for different aspects of perinatal derivatives (PnD) research, such as providing inputs for future standards for the processing, *in vitro* characterization and clinical application of perinatal cells and their secretome, will be fundamental to address this challenge.

Albeit cell heterogeneity doesn’t seem to be a *sine qua non-condition* for EV efficacy as demonstrated by many preclinical studies and initial clinical trials. Rather, standardizing EVs and understanding heterogeneity is crucial to fine-tune EV preparations for specific therapeutic applications and to select EVs that will provide an optimal response to the disease.

## Author Contributions

AC, APap, AM, APas, and ARS: writing – original draft preparation. FRS, ARS, and OP: writing – review and editing. OP: supervision and final approval of the manuscript. All authors contributed to the article and approved the submitted version.

## Conflict of Interest

The authors declare that the research was conducted in the absence of any commercial or financial relationships that could be construed as a potential conflict of interest.
